# Supplementary material for: Are carbon emissions trading and green financial instruments synergistic? -Comprehensive quantitative research based on content analysis
Source: PLoS One. 2024 Mar 7;19(3):e0298601. doi: 10.1371/journal.pone.0298601 (PMC10919601; doi:10.1371/journal.pone.0298601)
Supplement: S1 Data — (ZIP) [file pone.0298601.s001.zip › Data/地级市Z_F工作报告.zip!/╜¡╦╒/─╧═¿╩╨/└┤╫╘╣1⁄2╓┌║┼í╛┬φ┐╦╩2╛▌═°í┐_2019.docx]

各位代表：

现在，我代表市人民政府向大会作工作报告，请予审议，并请政协委员和其他列席人员提出意见。

一、2018年工作回顾

过去的一年，我们认真学习贯彻习近平新时代中国特色社会主义思想和党的十九大精神，以纪念改革开放40周年为动力，按照省委、省政府赋予南通争当“一个龙头、三个先锋”的新定位新使命，在市委的正确领导下，扎实推动“六个高质量”发展，统筹做好各方面工作，较好完成全年目标任务。地区生产总值8427亿元，增长7.2%；一般公共预算收入606.2亿元；全社会研发投入占地区生产总值比重2.67%；固定资产投资增长8.8%，社会消费品零售总额增长9%，进出口总额增长7.7%；城镇、农村居民人均可支配收入分别为46321元、22369元，分别增长8.3%、9.3%；居民消费价格涨幅2.3%；城镇登记失业率1.78%，新增就业10.4万人；完成省下达节能减排任务。

（一）突出提质增效，经济发展稳中有进。深化供给侧结构性改革，强化政策举措落实，保持经济平稳健康发展。优化实体经济环境。坚持降成本、优环境、强服务多管齐下，出台促进民营经济高质量发展“10条”措施，完善电力直接交易、工业用地先租后让等制度，开辟中小企业转贷、投贷联动、绿色债券等融资新渠道，新增直接融资451亿元、上市公司3家，小微企业新增贷款占比31.1%。围绕做大做强，实施支持企业投资、技改等“24条”措施，推进“5215”工业大企业培育计划，评选表彰改革开放40年优秀民营企业家，新增主营业务收入500亿元和100亿元企业各1家，新增全国制造业单项冠军4家。强化质量和效益导向，优化开发园区产业用地管理，建立工业企业绩效综合评价平台。推进产业转型升级。加快培育先进制造业集群，“3+3”重点产业产值增长13%。着力推动制造业智能化、服务化，加强质量强市建设，新增国家智能制造和“两化”融合试点企业4家、省级生产性服务业集聚示范区4家。大力发展装配式建筑、绿色建筑、智能建筑，建筑业总产值超过8000亿元。协同扩大内外需求。持续加大有效投入，省、市级重大项目完成投资超千亿元。实施支持消费结构升级等政策措施，新增省级品牌消费集聚区9家。积极应对贸易摩擦，引导有条件的企业向内外贸一体化转型，推动外贸集聚区、跨境贸易示范区等建设，提升口岸贸易便利化水平。

（二）突出精准施策，三大攻坚成效明显。健全体制、创新举措，着力突破难点问题和薄弱环节。打好污染防治攻坚战。整合设立市打好污染防治攻坚战指挥部，健全四级污染防治工作体系。以中央环保督察“回头看”、国家海洋督察、长江经济带生态环保审计等反馈意见整改为重点，扎实解决环境突出问题。制定碧水、蓝天保卫战三年行动实施方案，全力推进大整治，PM2.5平均浓度全省最低，优良天数比例全省最高。抓好水务一体化改革、畜禽和南美白对虾养殖污染治理等重点工作，新建污水管网290公里。完善化工产业导向目录，关闭小化工等“散乱污”企业220家。出台长江经济带生态环境保护规划，完成沿江非法码头整治后的复绿工作。成立生态环保执法司法联动中心，实施环保总监、生态环境损害赔偿等制度。“利剑斩污”专项行动成效明显，全社会治污合力进一步增强。打好精准脱贫攻坚战。落实精准脱贫三年行动计划，健全农村低保与扶贫开发相互衔接和健康扶贫等机制，建档立卡低收入人口脱贫率超过80%，经济薄弱村转化工作成效明显。与陕西汉中的扶贫协作、与新疆伊宁和青海贵德的对口支援任务全面落实。打好防范化解重大风险攻坚战。建立健全防范化解政府隐性债务风险的制度与政策体系，加强政府投资项目资金源头管控，推进融资平台公司市场化转型，政府性债务规模、债务率实现双下降。全面启动三年处非攻坚战，有效防控非法集资和互联网金融等风险。

（三）突出创新驱动，发展动能加快转换。围绕推动科技与产业结合，优化创新环境，提高创新能力。强化企业创新主体地位。落实省科技改革“30条”，出台支持产学研合同研发、重大科技专项配套补助等系列政策，高新技术企业总数超过1300家，新增省级以上企业技术中心30家、院士工作站6家。制定实施人才安居工程的意见，新增省“双创”人才57名，连续三年位居全省第二。实现国家级知识产权强县工程试点（示范）全覆盖，万人发明专利拥有量增长14%。强化区域创新平台建设。出台中创区专项政策，科创中心一期开工，4个院士团队、10家高校院所签订落户协议。发展船舶海工、先进通信、新材料等重大创新平台，启动国家技术转移东部中心南通分中心建设。打造“双创”全过程服务链，新增省级众创社区3家。强化新业态新模式培育。推进新一代信息技术产业发展行动，设立新一代信息技术产业专项资金，新落户智能芯片、半导体、大数据等领域亿元以上项目46个，城域网出口带宽突破11T。积极培育新增长点，电子商务交易额、服务外包执行额分别增长27.2%和50%。

（四）突出项目牵引，江海联动加速布局。抢抓国家、省支持通州湾海港建设等重大机遇，加强对港口、产业、集疏运体系的系统规划建设。深化港口一体化改革。编制形成《提升南通港定位打造江苏新出海口的方案》，以更高标准推进沿江沿海港口建设。通海港区一期开港运营，狼山集装箱港区完成搬迁。通州湾海港建设前期工作加快推进，洋口港国家级LNG产供储销体系枢纽点建设取得成效，吕四港区10万吨级进港航道二期疏浚工程完工。全面推进大交通建设。完善综合交通运输体系发展规划，被列为港口型、商贸服务型国家物流枢纽承载城市。南通新机场规划有序推进，北沿江高铁前期衔接取得重要进展，沪通长江大桥等工程快速实施，盐通铁路南通段完成动迁，宁启铁路二期建成通车。加快集聚重大产业项目。开展重点国家和地区“招商周”，组织沿海资源推介、日资企业恳谈等活动，新引进超亿美元外资项目38个、超50亿元内资项目8个。新开工10亿元以上项目100个，招商局豪华邮轮、恒科新材料二期、华峰超纤、金光纸业、中天精品钢、京东物流、宝能战略合作等重特大项目开工建设或成功签约。拓展陆海统筹开发开放。深入推动陆海统筹发展综合改革，江海资源配置进一步优化。深化上海“北大门”建设，产业、交通、科技、旅游等对接互动明显加强，承接亿元以上产业项目超过240个，促成产学研合作项目120个。以“一带一路”为重点开拓国际市场，新增境外投资项目68个。注册外资实际到账25亿美元。新增省级开发区（高新区）5家。

（五）突出统筹协调，城乡建设更有质量。编制完善2035城市总体规划，推动“四化”同步，城乡发展协调性进一步提升。加强中心城市建设和管理。中创区“一带四中心”建设全面实施，国家级五山森林公园获批，濠河风景区整治提升工程启动，获国家森林城市、省生态园林城市称号。轨道交通1号线进入主体施工阶段，2号线开工建设，啬园路隧道主体贯通，机场大道高架主线通车。推出以智能化、便捷化为重点的公交惠民“八项举措”。开展城市“微治理”三年行动，“八整治八提升”扎实推进。实施乡村振兴战略。编制战略规划，出台实施意见，抓好乡村振兴重点工程。“三个全覆盖”试点持续突破，新型合作农场建设稳步推进，新增高标准农田53.3万亩、规模经营主体952个。促进农村一二三产业融合发展，新增省级农业产业化龙头企业8家、国家级农民专业合作社示范社17家。推进农村人居环境整治三年行动，15个镇、25个村获省生态文明建设示范称号。夯实城乡协调发展基础。制定加快新型城镇化建设行动方案，3个经济发达镇行政管理体制改革试点启动实施。土地节约集约利用获国务院通报表扬。农村承包地确权登记颁证通过省级验收，集体产权制度改革完成清产核资。统筹城乡基础设施建设，“四好农村路”创建全省示范，九圩港复线船闸和提水泵站建成运行，通吕运河水利枢纽开工建设。

（六）突出共建共享，人民生活持续改善。顺应群众对美好生活的向往，提高民生保障实效，完成50项重点民生实事。深入推动富民增收。落实“富民50条”政策措施，打造就业协作服务平台和市级创业示范基地，实施以新生代农民工为重点的“双提升”计划，发放创业担保贷款2.3亿元、稳岗补贴6355万元，扶持群众创业1.9万人。提升基本公共服务水平。推进教育优质均衡发展，增加学前教育学位8190个、义务教育学位10530个和普通高中招生计划4440个，情境教育入围中国质量奖提名奖。实施健康南通建设三年行动计划，公立医院患者满意度保持全省前列，获评健康中国年度标志城市。出台市区因病致贫救助办法，城乡居民基础养老金、城乡低保、被征地农民保障等标准继续提高。国家级居家和社区养老服务改革试点工作获评优秀等次。房地产市场基本稳定。南通大剧院、美术馆等重大文体设施开工建设，全市镇街文化站、村（社区）综合性文化服务中心实现全覆盖，张謇题材系列文艺作品产生良好反响。世界体育冠军增至20位，支云足球俱乐部成功“冲甲”。大力创新社会治理。严格落实综治、法治、信访和国家安全工作责任制，推进扫黑除恶专项斗争，创新“网格化+信息化+精细化”社会治理体系，省级法治县（市）区创建先进集体实现“满堂红”，公众安全感、法治建设满意度持续位居全省前列。安全生产和食品药品监管工作进一步加强，民族、宗教等工作取得新成效。

过去的一年，我们把坚持和加强党的全面领导贯穿政府工作各方面、全过程，积极开展解放思想大讨论，着力打造现代政府。全面加强政府系统党的建设。坚持以习近平新时代中国特色社会主义思想定向领航，筑牢“四个意识”、践行“两个维护”。严格执行中央八项规定和实施细则精神，从严落实党风廉政建设责任制。健全市政府党组工作制度，完善推进、协调、督查机制，不折不扣落实上级决策部署和市委要求。持续提升行政效能。狠抓“放管服”改革，强化政府信息化、政务智能化建设，打造“不见面审批、零缺陷服务、精准化监管”品牌，在全省第二轮“双创”环境评价中蝉联第一。推动“3550”常态化，政务服务一张网贯通93个乡镇、1795个村居，“南通百通”APP上线运行。县域综合执法改革被国务院第五次大督查列为典型经验并通报表扬，“双随机一公开”实现全覆盖。“走帮服”活动深入开展。大力促进勤政廉政。落实省、市委“三项机制”要求，引导激励公职人员担当作为。完善法治政府建设督察推进机制，配合做好烟花爆竹燃放管理、畜禽养殖污染防治等立法工作，全面推开行政执法三项制度。认真办好市人大代表议案、市政协建议案，完成市人大代表建议、政协提案办理任务。自觉落实中央、省委巡视和市委巡察整改意见，主动接受各方监督，干部廉洁自律意识进一步增强。

各位代表，过去一年的成绩凝聚着全市人民的汗水与智慧，离不开各方面的关心和支持。在此，我代表市人民政府，向全市人民，向人大代表、政协委员和离退休老同志，向市各民主党派、工商联、无党派人士、各人民团体和各界人士，向驻通人民解放军、武警官兵、公安干警和消防救援指战员及部省驻通单位，向关心支持南通发展的海内外朋友，致以崇高敬意和衷心感谢！

我们也清醒看到，全市经济社会发展还面临不少困难和挑战，政府工作还有许多不足。做大总量、提升质量仍是现阶段的主要任务，科技创新能力不强，龙头型企业还不多。实体经济生产经营压力依然较大，支持企业发展的政策举措需要完善和深化。生态环境保护任务艰巨，水环境质量亟待提升，污染防治能力建设相对滞后。城乡融合发展机制不够健全，中心城市首位度、竞争力有待增强。教育、医疗、养老等公共服务供给的结构性矛盾比较突出，市场化手段运用不足，与人民群众的期盼有较大距离。少数政府工作人员服务能力、担当意识不够，“慵懒散”和不作为、慢作为现象还不同程度存在。对此，我们将直面问题，敢于担当，切实加以解决。

二、2019年目标任务

今年是新中国成立70周年，也是决胜高水平全面建成小康社会的关键之年。世界经济环境复杂严峻，国内经济运行稳中有变。 我们不仅要看到前进中的困难，更要看到我国发展仍处于并将长期处于重要战略机遇期。有以习近平同志为核心的党中央坚强领导，我们就一定能保持信心和定力，战胜任何风险与挑战。特别是随着“一带一路”建设深入推进，长三角区域一体化发展上升为国家战略，通州湾被赋予建设长江经济带战略支点的新要求，省委、省政府支持我市打造江苏新出海口，南通集多重国家和区域战略于一身，迎来改革开放以来又一次重大发展机遇，进入新时代加速发展、追赶超越的“窗口期”。我们必须准确把握南通正处于工业化中后期的阶段实际，切实增强“解放思想、走在前列”的使命感与危机感，抢抓机遇，守住底线，能快则快，做大总量，为新一轮发展积蓄动能和势能，为迈向“万亿俱乐部”打下扎实基础，不断开创“强富美高”新南通建设新局面。

做好今年政府工作，要坚持以习近平新时代中国特色社会主义思想为指导，全面贯彻落实中央、省和市委各项部署要求，统筹推进“五位一体”总体布局，协调推进“四个全面”战略布局，坚持稳中求进工作总基调，坚持新发展理念，坚持推动高质量发展，坚持以供给侧结构性改革为主线，坚持深化市场化改革、扩大高水平开放，以建设长江经济带战略支点和上海大都市北翼门户城市为重点，以开展重特大项目落地落实年活动为抓手，继续打好三大攻坚战，着力提升产业发展、城市服务、交通枢纽能级，扎实做好稳增长、促改革、调结构、惠民生、防风险工作，争当“一个龙头、三个先锋”，为高水平全面建成小康社会收官打下决定性基础，以优异成绩庆祝中华人民共和国成立70周年。

今年全市经济社会发展的主要预期目标是：地区生产总值增长7%以上，一般公共预算收入增长5%以上，全社会研发投入占地区生产总值比重2.7%以上；固定资产投资增长8%以上，社会消费品零售总额增长9%左右，进出口总额增长4%以上；城乡居民收入增长与经济增长同步，居民消费价格涨幅3%左右，城镇登记失业率控制在3%以内、新增就业8万人以上；生态环境质量持续改善，单位生产总值能耗、主要污染物排放进一步降低。

工作中，要突出“六个稳”、“六个高质量”和打造“三大枢纽”、提升“三大能级”等重点，统筹抓好十个方面：

（一）坚持产业强市不动摇，加快发展先进制造业。牢固树立制造业总量意识，大力提升产业能级，夯实高质量发展根基。

聚焦地标性产业和企业培育。落实培育先进制造业集群的实施意见，提升“3+3+N”重点产业链水平，规模以上工业总产值达1.5万亿元左右。推动船舶海工拓展关键配套和专用设备等领域，加快家纺向个性化设计、定制化生产转型，支持光通信、集成电路集聚上下游项目，做优新材料细分行业，打造新能源汽车全产业链。做大专业化园区，培育生物医药、石墨烯、工业机器人、氢能源等特色产业。发挥新一代信息技术产业专项资金带动效应，引进智能芯片、半导体等领域的领军型企业、高精尖项目。推动智慧建筑产业化项目落地实施。深入推进“5215”工业大企业和“专精特新”企业培育计划，力争新增百亿级企业2家。

发展智能制造和服务型产业。加快大数据、人工智能与实体经济深度融合，推进工业互联网平台建设，新增“上云”企业2000家，建成地理、法人等五大基础数据库和全市统一的数据共享交换平台。深入实施企业智能化改造三年行动计划，创建智能制造示范区，新增省级以上智能车间（示范工厂）10个。推动现代服务业与先进制造业深度融合，培育供应链物流等新业态，打造上海金融后台服务基地，壮大服务外包产业规模。实施消费升级行动计划，加快把旅游休闲打造成支柱产业，发展教育健康、育幼养老、文化体育等服务业，挖掘信息消费、品质消费等潜力。

强化项目建设和招大引强。开展重特大项目落地落实年活动，加强对8个在手百亿元以上产业项目的精准协调服务。完善跟踪推进机制，抓好省级重大项目和市级“双百工程”项目。狠抓招商引资不放松，聚焦日韩、欧美、新加坡、港台和深沪等重点地区，统筹组织招商活动，主攻投资体量大、科技含量高、带动能力强的项目，提高精准化、专业化和产业链招商水平，力争新引进超亿美元外资项目40个、超50亿元内资项目10个。

（二）健全“双创”促进体系，加快打造科技创新高地。坚持“三个第一”，积极承接上海科创资源溢出效应，广泛集聚国内外创新要素，建设长三角特色产业科技创新基地。

支持创新主体发展。深入实施高新技术企业培育工程，高新技术产业产值占规上工业比重达50%左右。大力推进以企业为主体、市场为导向、产学研深度融合的技术创新，推动面广量大的中小企业广泛开展合同研发，实施产学研合作项目1000项，新增省级以上研发机构55家。深入推进标准化综合改革和质量提升行动，主导或参与制（修）订各级标准50项。用好江海英才创业周等平台聚才引才，新增省“双创”人才30名以上。深入实施“通籍人才归雁计划”，吸引更多高校毕业生留通返乡就业创业。建设高素质产业工人队伍，新增高技能人才1.4万名。

深化创新平台建设。加快推进中创区与大院大所、知名高校和企业的合作项目，大力引进科创类项目和创新型人才，争取国家、省重大科技项目。发挥高新区科技创新主阵地作用，支持创建省级以上创新型特色园区、知识产权试点（示范）园区。落实沪通科技创新全面战略合作协议，共建一批科技合作园区和公共服务平台。加快“3+3”产业省级以上研发机构建设，创建海洋装备和高技术船舶等省级制造业创新中心。推动众创空间专业化、品牌化发展，打造特色众创社区。

优化创新创业生态。发挥南通大学和南通高研院、产研院等作用，招引科研院所和企业研发中心、新型研发等各类机构，在南通开展创新创业和科技成果转化。加快引入风投、创投基金，优化产业基金运作模式，引导和扶持创新型中小企业落户并快速成长。抢抓上交所设立科创板的机遇，鼓励和服务更多科技企业挂牌上市、发展壮大。实施人才安居工程，构建更具吸引力的人才政策，为各类人才创新创业提供全链条服务。推进知识产权强企行动，创建国家知识产权强市，万人发明专利拥有量达29件。

（三）推动江海联动发展，加快建设战略支点。发挥沿江沿海独特资源优势，挖掘江海联动最大潜能，更大力度推进港产城融合，提高南通在国家和区域发展战略中的地位。

推进新出海口建设。优化南通港功能定位，加快港口一体化改革和江海资源整合，构建与新出海口相匹配的发展格局。按国际先进标准加快推进通州湾港区规划建设，开工一港池进港航道和起步码头工程，深化网仓洪20万吨级航道升等和冷家沙30万吨级航道前期工作。抓好洋口港区液化品码头二期、吕四港区10万吨通用码头、横港沙现代化散货码头等建设。加强与长江中上游重点城市合作，增强港口物流、中转、贸易等服务功能。

提升交通枢纽能级。以打造江海联运枢纽为目标，开工建设通海港区至通州湾港区疏港铁路一期、通扬线航道市区段等工程，做好洋吕铁路、新江海河、东灶新河等项目前期工作。以打造空铁枢纽为目标，深化南通新机场和通苏嘉甬铁路等前期工作，推动北沿江高铁南通段尽早开工，加快沪通铁路、盐通铁路建设。着力构建“一环接苏南、二环连浦西、三环通浦东”的高速路网，实施宁通高速扩容、绕城高速、苏通二通道等项目，海启高速、锡通高速北接线建成通车。以打造互联网枢纽为目标，力争成为首批5G商用城市，实现与北京等重点互联网城市直联。

培育沿海临港高端绿色产业。积极招引大进大出、大吞吐量、利用港口岸线的大项目，加快构建沿海现代产业体系。全力推进通州湾精品钢生产基地建设，培育特种钢铁产业集群。积极发展高技术、高附加值、高环保标准的石化新材料产业。依托洋口港LNG能源岛建设一体化配套产业园区，推进LNG海上过驳和已批海上风电项目建设。以吕四港为重点布局海产品和粮油食品储运加工项目。培育生物医药、航运服务和滨海旅游等新兴产业，促进海洋渔业向远洋捕捞及加工贸易一体化拓展。

（四）推动重点领域改革，加快创新体制机制。落实供给侧结构性改革“巩固、增强、提升、畅通”的八字方针，回应企业和群众关切，提高政府服务效能，激发发展动力和活力。

深化“放管服”改革。拓展“3550”改革成果，打造全国一流的营商环境。推进“证照分离”特别是“照后减证”，推广数字化审图、区域评估和企业投资项目承诺制，提升审批服务标准化便民化水平。深化“互联网+政务服务”，推进政务服务事项全部具备“不见面”条件，优化“南通百通”APP服务功能，让企业和群众办事像网购一样方便。做实各级政务大厅“一站式”服务功能，健全帮办代办、“全科窗口”服务机制，实现线上“一网通办”与线下“只进一扇门”集成融合。深化“双随机一公开”，推行信用承诺、审慎监管等新型监管模式。

激发微观主体活力。推进投融资体制改革，放宽市场准入门槛，积极推动政府和社会资本合作，发挥企业主观能动性。加快要素市场建设，深化陆海统筹土地利用和管理改革。完善工业企业资源利用综合评价机制，推动“僵尸企业”处置和低端低效产能退出，促进正向激励和优胜劣汰。构建市场监管一体化平台，完善公平竞争、稳定透明的法治化环境。深化国资国企改革，推动国有资本整合重组、优化布局，稳步推进混合所有制改革。

推动民营经济发展壮大。全面落实促进民营经济高质量发展的政策措施，深入开展“南通企业家日”等活动，更大力度保护和支持民营企业，让企业家政治上更受重视、发展上更有活力、权益上更有保障，在南通扎根发展、健康成长。切实缓解民营企业和小微企业融资难融资贵等问题，设立纾困基金，做大政府融资担保规模，支持金融机构创新转贷续贷、投贷联动等业务。全面落实用地、房产、物流等税费减免和社保降费率政策，降低企业成本130亿元以上。进一步畅通政企沟通渠道，发挥商会和行业协会作用，提高涉企服务针对性、有效性。

（五）发挥通江达海优势，加快完善高水平开放体系。以更宽的视野融入国家开放大布局，全面提高经济国际化水平。

扩大高水平双向开放。主动适应世界经贸格局的重大变化，以加强政府服务引导企业练好内功，深度融入国际产业链、价值链。推动稳外资与稳外贸互动并进，组织好海外南通名品贸易促进活动。推进市场采购贸易方式向内外贸结合发展，壮大跨境电商、外贸综合服务、航空维修等新业态。落实服务外包提档升级政策，大力发展服务贸易。积极参与“一带一路”建设，支持有实力的企业到沿线国家和地区布局，推动对外工程承包转型升级、境外产业集聚区模式更新，新增境外投资8亿美元。统筹综保区区港联动、机场监管场所和指定口岸建设。落实长三角一体化三年行动计划任务，加强规划衔接和项目争取。积极对接上海大都市圈空间协同规划，在互动融合中争取主动、增创优势。加大与上海在产业、科技、人才等方面的对接力度，力争引进亿元以上产业项目200个、产学研合作项目100个。用好南通籍在外经商人员多的优势，既加强“走出去”服务，又鼓励回乡创业，支持通商群体在扩大双向开放中发挥更大作用。

打造高水平开发园区。充分发挥开发园区在加快发展中的主力军作用，力争省级以上开发园区经济总量占全市比重提高5个百分点左右。加强企业化专业化招商队伍建设，推行市场化招商机制。坚持“亩产论英雄”，严格执行亩均投资、产出、税收和土地价格、招商成本等要求，支持建设多层标准厂房、定制厂房，提高开发园区投资强度和发展效益。围绕招商引资、经济发展、争先晋位、债务化解等重点，对省级以上开发园区实施科学精准的绩效考核，倒逼发展水平提升。复制推广上海自贸区、苏南自主创新示范区等体制机制创新经验，支持苏通科技产业园等跨江合作园区探索区域协同融合发展新模式。

（六）深化“三城同创”，加快提升城市品质和服务能级。围绕办好2019中国森林旅游节，实施“一节一带五片区”重点工程和城建重点项目，推进国家生态园林城市创建和花园城市建设，提高中心城区首位度和竞争力。

优化城市空间布局。继续完善2035城市总体规划，建好用好“多规合一”信息平台。统筹推进滨江主城、通州湾新城、启东生命健康科技城建设，引导中心城区提高经济密度、建筑密度和人口密度，促进各县（市）、各组团各展所长、互动并进。深化“城市双修”和城市设计试点，加强特色空间、景观、风貌打造以及天际线和色彩控制，塑造城市“山水城林”特色。

提高建设和管理水平。扎实推进濠河风景区整治提升，抓好水环境整治、路网优化、绿化景观、街景提升等重点项目，初步实现步道成环、水景开敞、四季有景、文化彰显。加快实施中创区配套项目，尽快展现区域形象。推动五山及沿江片区生态修复和基础设施、功能性项目建设。统筹五水片区城市设计和建设，打造城市西北部商业副中心。启动铁路西站片区核心区综合开发。抓好轨道交通1、2号线建设，加快骨干道路“白改黑”和拓宽改造、绿化提升。开展“微治理”集中攻坚大会战，加强对城市出入口、重点景区、主要道路沿线等环境整治，持续开展户外广告、违法建设等专项治理，继续推进“城中村”改造、老旧小区整治。加快智慧城市建设，打造一批应用示范项目。

大力发展城区经济。推进集研发、制造、商住等功能于一体的都市工业综合体建设，招引科创型、成长型企业落户，支持发展高端制造业，壮大科创经济、促进产业集聚。加快发展楼宇经济，强化运营招商和楼宇服务，打造一批以金融、外贸、文创、设计、功能性总部等为特色的主题楼宇。积极壮大工业设计、检验检测、电子商务、快递物流等都市型产业，培育和丰富贴近市民需求的新业态，打造共享经济、生态经济等新增长点。

（七）统筹城乡融合发展，加快实施乡村振兴战略。坚持农业农村优先发展，提高新时代“三农”工作针对性。

推进现代农业发展。深化农业供给侧结构性改革，推动村集体建立股份制专业合作社并领办新型合作农场，放大“三个全覆盖”试点效应，培育新型农业经营主体和新型职业农民，提升农业规模化水平和农民组织化程度，建成新型合作农场的村达到150个以上，新建高标准农田40万亩以上。加快国家现代农业示范区和农业科技园建设，壮大“互联网+农业”、特色农产品精深加工和休闲观光农业等新业态新模式，大力发展一县一业、一镇一特、一村一品。提高粮食安全保障水平和“菜篮子”保供能力，确保农产品质量安全。

提升农村生活品质。实施“百村示范、千村整治”工程，切实改善农村人居环境。统筹推进城乡“厕所革命”，完善城乡垃圾分类分拣和转运处置体系，因地制宜布局符合农村特点的污水、垃圾收集处理装置，实施农村环境“五位一体”长效管护。推动农村基础设施提档升级，全面推进“四好农村路”建设。扎实抓好特色田园乡村试点和美丽乡村建设。

健全城乡融合发展体制机制。编制县域乡村建设规划，开展村庄规划实施评估，持续推进城乡产业布局、基础设施、公共服务等一体化，引导更多要素向农村流动。推动新一轮经济发达镇行政管理体制改革，培育一批产城融合的特色小镇。深化农村改革，拓展土地承包经营权确权登记成果应用，探索宅基地“三权分置”有效实现形式，加快集体产权制度改革。

（八）深入推进污染防治攻坚，加快改善环境质量。持续打好碧水、蓝天、净土保卫战，让广大群众感受到环境治理的成效。

加强系统化治污。聚力攻坚水环境治理，严格实施“河长制”“断面长制”，以通启、通吕、如泰运河等流域为重点，落实面源污染治理、生猪养殖管理、企业达标排放、活水畅流等综合措施，确保地表水省考以上断面优良比例达71%；实施中心城区水质提升工程，以水系为脉络、黑臭水体为重点开展整体治理，力争濠河及周边主要河道达到Ⅲ类水标准；继续推进县级以上集中式饮用水源地水质达标建设。抓好扬尘、餐饮油烟、柴油货车等污染治理以及重点行业领域节能减排，保持空气质量全省领先。开展土壤污染修复和重金属防控区专项整治，保障土壤环境安全。坚持产业生态化与生态产业化并举，完成化工园区和“散乱污”企业整治，指导帮助企业制定环境治理解决方案，提高污染源头防控水平。

推进污染防治能力建设。探索多元化运作模式，补好环境基础设施短板。加快水务一体化改革，实现供水、排水和污水处理一体化，乡镇污水处理厂负荷率、建成区管网覆盖率均超过70%。制定固废基础设施布局规划，推进危废焚烧、飞灰填埋等集中处置项目，危废本地消纳率超过60%。实施园区环境治理工程，提升污染物收集和处置、能源清洁化利用、环境监测监控能力。

健全生态文明体制机制。严格落实环境保护“党政同责、一岗双责”，对生态环境损害实行终身追责。推进环境质量和污染排放总量双控管理，完善排污权有偿取得和交易、区域生态补偿等机制，实施与污染物排放总量挂钩的财政政策。健全网格化监管体系，落实重点排污单位环保总监制度，深入开展“利剑斩污”专项行动，全面推动环保行政执法和刑事司法有效衔接。

强化生态修复和保护。坚持共抓大保护、不搞大开发，深入落实《沿江经济带绿色发展重点任务行动方案》，编制“三线一单”，推动“三水共治”，深化长江岸线综合治理和海岸线整治修复，强化入海河流、排污口整治，推进河湖违法圈圩和违法建设专项治理，确保主要入江支流消除劣Ⅴ类，近岸海域水质保持稳定。加强沿江沿海生态廊道、生态保护引领区建设和生物多样性保护，实施长青沙、开沙岛等沿江区域绿化工程，争创全国绿化模范城市，林木覆盖率稳定在24%以上。

（九）弘扬江海特色文化，加快提高文化软实力。坚定文化自信、打造文化标识，更好发挥文化的凝聚和引领作用。

深化文明城市建设。深入开展中国特色社会主义和中国梦宣传教育，弘扬“包容会通、敢为人先”的城市精神，使全市人民在理想信念、价值理念和道德观念上紧紧团结在一起。推进新时代文明实践中心建设试点，更好引导群众、成风化俗。推动新一轮文明城市创建和全域创建，放大精神文明“南通现象”。深化诚信南通和社会信用体系建设，推动志愿服务制度化。繁荣发展哲学社会科学。全面落实意识形态工作责任制。

传承创新传统文化。抓好濠河风景区历史文化提升和环濠河博物馆群功能提升，加强寺街西南营等历史文化街区保护。制定传统工艺振兴计划。推动大运河文化带南通段建设。推进具有江海文化特征的艺术精品创作，聚焦非遗、杂技、话剧、足球、老电影等优势项目，做精做优特色文体品牌。

促进文化事业和产业发展。推动现代公共文化服务体系建设，提升公共图书馆、文化馆和农家书屋等文化设施使用效率，办好重点文化品牌活动。壮大文化创意产业，推动文化与科技、金融、旅游、互联网等深度融合，实施50个重点产业项目，培育国有、民营文化龙头企业，做强文化产业示范园区（基地）。

（十）优化公共服务，加快推动富民惠民安民。多措并举促进公共服务标准化、均等化、多元化，继续为民办好46项实事，切实增强人民群众获得感、幸福感和安全感。

提高就业增收水平。深入实施富民增收五大行动计划，完善稳就业、促创业、防失业的政策体系。加大培训促进就业力度，帮扶高校毕业生、农民工、退役军人等1.5万名以上群众创业。落实个税专项附加扣除政策。健全劳动关系协调机制。继续实施重大疾病、意外伤害商业保险等健康扶贫政策，强化产业扶贫、结对帮扶，力争建档立卡低收入人口全部实现脱贫。大力支持92个经济薄弱村增加经营性收入。探索解决相对贫困的预警和长效机制。加强老区建设，继续做好扶贫协作和对口支援等工作。

完善社会保障体系。持续推动全民参保，确保五大保险参保率稳定在97.5%左右。稳步提高各类保障标准，扩大企业年金覆盖面。深入实施国家级照护保险试点。在近年来稳步推进通州区与主城区一体化的基础上，实现城乡居民养老、医疗保险全面接轨。推进医保支付方式改革。构建房地产市场健康发展长效机制，持续做好稳房价工作，加快推进市区危房解危，开展既有多层住宅加装电梯试点工作。完善社会救助、福利、慈善制度，健全残疾人关爱服务体系。积极发展妇女、儿童、青少年、关心下一代、老龄、红十字等事业。

加快教育优先发展和健康南通建设。制定实施《南通教育现代化2035》，加大全社会教育投入。扩大普惠性幼儿园覆盖面，创建国家级义务教育优质均衡县（市、区），推进高中阶段学校考试招生制度改革。深化职业教育校企合作、产教融合，办好特殊教育、继续教育。支持在通高校高水平发展，推动通师高专“升本”。加强师德师风建设，完善师资均衡配置机制。大力发展民办教育，积极引进优质教育资源，满足不同层次教育需求。深化公立医院改革，优化市区医疗资源布局。制定实施全科医生队伍建设三年行动计划，提升医联体建设和家庭医生签约服务水平，做实分级诊疗机制。

推动慢性病综合防控示范区建设，创建国家医疗健康大数据应用示范城市。扎实推进国家级居家和社区养老服务改革以及医养结合试点，提升护理型床位占比。深入实施全民健身五年计划，建设全国青少年校园足球改革试验区。

全力维护安全稳定。把守住底线作为能快则快、做大总量的前提，为发展营造更好环境。坚持“清、规、控、降、防”并举，有效防范化解政府隐性债务风险，加大非法集资等金融风险防控力度，确保不发生重大风险。扎实抓好扫黑除恶专项斗争、“雪亮工程”、信访标准化建设、网格化服务管理，强化犯罪打击、矛盾化解、源头防控，切实保障公共安全。加强社区共治和村民自治，规范发展社会组织，完成新一届村（居）委会换届选举，97%的城市社区、93%的农村社区达到省级和谐社区标准。推进安全生产领域改革发展，严格落实安全生产责任制，防范和遏制安全事故。强化食品药品安全监管。争创“七五”普法中期验收先进市。推进军民融合深度发展，做好国家安全、全民国防教育和退役军人事务、民族、宗教、档案、保密、地方志等工作。

三、加强政府自身建设

认真贯彻新时代党的建设总要求，强化“四个意识”、坚定“四个自信”、践行“两个维护”，更大力度推进政府治理体系和治理能力现代化，更强效能保障高质量发展。

（一）以机构改革深化职能转变。围绕打造政府和市场“双强引擎”，全面完成政府机构改革，构建系统完备、科学规范、运行有效的职能体系，推动职能整合、业务整合真正发生“化学反应”，加快建设人民满意的服务型政府、精简集约高效的有限型政府。进一步健全权力清单、负面清单、责任清单制度，最大限度简政放权、再造流程，充分激发市场活力和社会创造力。创新行政管理，严格行政问责，不断提升行政执行力和政府公信力。

（二）以依法行政规范政府行为。落实法治政府建设任务，推动重点领域立法，做好行政复议、应诉工作，强化规范性文件合法性审核。推进重大行政决策网上运行、流程监控和全过程记录。深化综合行政执法体制改革，严格执行行政执法三项制度。依法接受党内监督、人大法律和工作监督、政协民主监督以及国家监察、司法监督，办好人大代表议案、建议和政协建议案、提案。重视社会监督、舆论监督，加强审计监督，深化重大建设项目、公共资源配置、公益事业等领域政务公开。

（三）以能力建设引导担当实干。充分认清总量是奋斗出来的、发展是拼抢出来的，做努力奔跑、不负时代的实干者。锤炼干事创业本领。坚持用习近平新时代中国特色社会主义思想武装头脑、指导实践、推动工作，顺应高质量发展新形势新要求，运用系统化思维、注重规律性把握，全面增强“八个本领”，练就担负起新使命的宽肩膀、硬肩膀。增强改革创新意识。不为成绩所困，不为定势所累，坚决摒弃与新时代要求、新发展理念不相适应的传统思维和路径依赖。坚持目标导向、问题导向、结果导向，紧扣重点工作落实创新体制机制，真正用改革为发展打开一片新天地。弘扬务实苦干精神。深入落实“三项机制”，牢固树立重实绩的鲜明导向，敢对发展负责，善为人民尽责，让改革者轻装上阵、担当者脱颖而出、创业者大显身手，形成争先创优的浓厚氛围。大力倡导马上就办的作风，对定下来的事情雷厉风行、抓紧实施，对部署了的工作跟踪问效、一抓到底。

（四）以从严治政锻造过硬作风。严格落实全面从严治党主体责任和“一岗双责”，带头做到“五个过硬”，推动政府系统党风廉政建设向纵深发展。深入贯彻中央八项规定和实施细则精神以及省委、市委要求，集中整治形式主义、官僚主义，持之以恒纠正“四风”。坚持政府过“紧日子”，严控“三公”经费和一般性支出。深化重点领域和关键环节廉政风险防控，严厉整治发生在群众身边的腐败和作风问题，树立为民务实清廉的良好形象。

各位代表，新时代是奋斗者的时代，也是成就伟业的时代。让我们更加紧密地团结在以习近平同志为核心的党中央周围，在省委、省政府和市委的坚强领导下，凝心聚力，砥砺奋进，为推动高质量发展走在前列、谱写中国梦南通新篇章而努力奋斗！
